# A Novel Cause of Biliary Peritonitis after Endoscopic Retrograde Cholangiopancreatography: Case Report and Literature Review

**DOI:** 10.1155/2021/3814080

**Published:** 2021-10-21

**Authors:** Andrija Karačić, Paula Batur, Domagoj Štritof, Taro Fukui, Branko Bakula, Inka Kekez

**Affiliations:** ^1^Department of General Surgery, Clinical Hospital Sveti Duh, Zagreb, Croatia; ^2^School of Medicine, University of Zagreb, Zagreb, Croatia; ^3^Department of Surgery, Saitama Medical Center, Jichi Medical University, Saitama, Japan; ^4^Department of Radiology, Clinical Hospital Sveti Duh, Zagreb, Croatia

## Abstract

**Background:**

Endoscopic retrograde cholangiopancreatography (ERCP) can lead to several complications such as duodenal or bile duct perforation. The incidence of pneumoperitoneum post-ERCP is rarely seen (<1%) and is associated with perforations of the duodenum or common bile duct in therapeutic ERCP after sphincterotomy. In this case, we disclose a novel cause of biliary peritonitis after ERCP. *Case Presentation*. A 65-year-old man presented with abdominal pain and distended abdomen after uneventful ERCP with sphincterotomy. An abdominal computed tomography (CT) was performed whose finding indicated duodenal perforation. The patient was rushed to an emergency laparotomy where only a rupture of an otherwise normal subcapsular intrahepatic bile duct was found. The surrounding liver parenchyma was healthy. The cause of this condition was probably post-ERCP pneumobilia and the increase of pressure in the biliary tract.

**Conclusions:**

This is the first case in literature describing the rupture of a subcapsular healthy bile duct as cause of biliary peritonitis after ERCP. This case also suggests that in the management of post-ERCP complications, the cooperation of radiologists and surgeons is vital for the patient's wellbeing.

## 1. Background

Endoscopic retrograde cholangiopancreatography (ERCP) has, in its more than 50 years of clinical practice, been proven to be a relatively safe procedure not only in the diagnosis but also treatment of biliary and pancreatic diseases [1]. The main advantage of ERCP above its alternative, hepatobiliary surgery, is lower morbidity and mortality [2]. The most common complications of ERCP are infections such as cholangitis and pancreatitis, bleeding, or intestinal perforation [3]. Some therapeutic additional options, especially sphincterotomy, increase the risk of intestinal perforation [4]. Iatrogenic post-ERCP duodenal perforation is regarded as a common complication, while intrahepatic bile duct ruptures are rare and have been associated in case reports with surgically damaged liver tissue, liver metastasis, and abscesses [5, 6]. In our case report, the radiologist found the biliary peritonitis to be a consequence of duodenal perforation; however, the surgical team intraoperatively identified a ruptured, but otherwise, healthy subcapsular hepatic bile duct as the cause of the biliary peritonitis. A literature review was performed for similar cases, and no case of healthy hepatic bile duct rupture causing biliary peritonitis after ERCP has been reported. Written informed consent has been given by the patient. The following case report has been reported in line with the SCARE criteria [7].

## 2. Case Presentation

A 65-year-old male was referred to the gastroenterology department with obstructive icterus. His medical history was remarkable for arterial hypertension, gastric ulcer, and chronic gastritis, diverticulosis of the colon and sideropenic anemia. He presented with epigastric abdominal pain, nausea, and heartburn. His physical examination revealed icterus of the skin and conjunctiva. His bilirubin was found to be elevated (107 *μ*mol/l). AST and ALT were 114 IU/l and 217 IU/l, respectively. The WBC count was 10.42 × 10^3^/mm [3], and segments were 24.7%.

**Figure 1:**
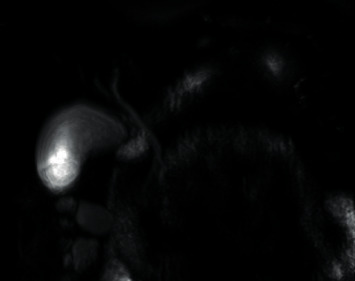
MRCP—choledocholithiasis; no signs of common bile duct dilation and normal intrahepatic bile ducts.

Ultrasound revealed an elongated gallbladder (10.5 cm) filled with sludge and tiny concrements, and the common bile duct was normal in diameter, without visible intraluminal substrate. The liver was slightly enlarged with hyperechogenic parenchyma diffusely. Computed tomography (CT) of the abdomen showed normal liver structure and an enlarged gallbladder without signs of pathological changes in the wall or lumen. MRCP showed choledocholithiasis, no signs of common bile duct dilation, and normal intrahepatic bile ducts (Figure 1).

He underwent ERCP for choledocholithiasis. Access to the major papilla next to a periampullar diverticulum was obtained, and a widened common bile duct was shown with small concrements prepapillary. A sphincterotomy and concrement extraction with a balloon was performed. The procedure passed without complications.

**Figure 2:**
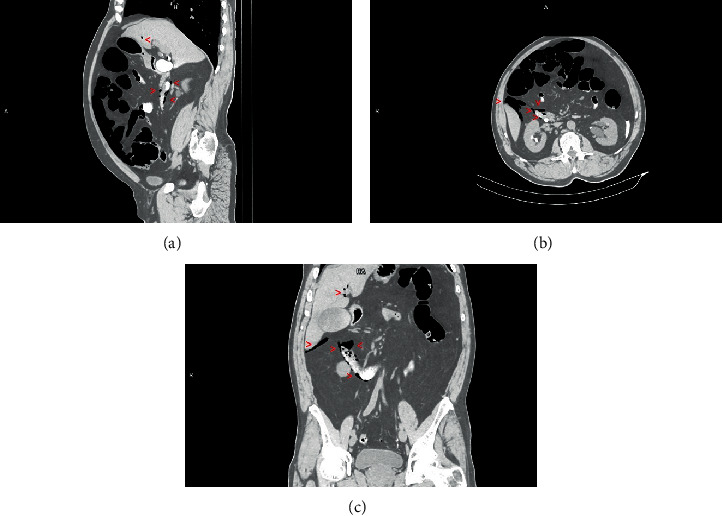
CT—free air around the duodenum in level of the papilla around the liver and visible retroperitoneum around the liver.

**Figure 3:**
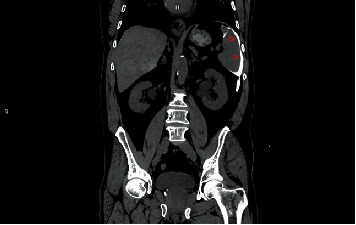
CT–free contrast around the gastric fundus and spleen.

While waking up from the sedation, the patient immediately complained about abdominal pain. The gastroenterologist found the abdomen to be distended and referred the patient to an urgent CT of the abdomen. The WBC was 10.42 × 10^3^/mm [3]. On CT, free air was found around the duodenum in level of the papilla in the retroperitoneum and around the liver equaling pneumoperitoneum and pneumoretroperitoneum (Figure 2). Postprocedural pneumobilia in the gallbladder, cystic duct, common bile duct, and intrahepatic bile ducts was found. Free contrast was found around the gastric fundus (Figure 3), paracolical, and pelvis, and the radiologist suspected duodenal perforation.

**Figure 4:**
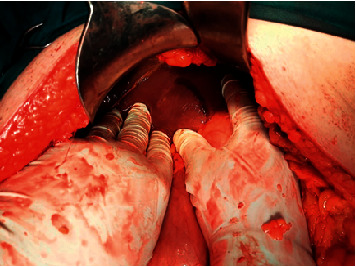
Intraoperative finding.

The patient was referred urgently to the general surgery department. The patient's abdomen was found to be distended with signs of peritonitis, no audible peristalsis. The patient was rushed to an emergency laparotomy. Intraoperative biliary peritonitis was found. After thorough exploration of the upper abdomen and kocherization of the duodenum and head of the pancreas, no perforation of the duodenum or common bile duct was found. Hence, retroperitoneal perforation as the source of the intraperitoneal biliary collection could be excluded. Only when removing the liver retractor on the anterior side of the III, liver segment ruptured, but otherwise, normally appearing bile duct was found with bile secretion into the peritoneal cavity (Figure 4). The defect was closed with sutures. A choledochotomy was performed, and a T-drainage positioned to facilitate the biliary drainage. Cholecystectomy, lymphadenectomy, and omentectomy were performed too. Since the site of the intrahepatic bile duct rupture was clearly seen, no indication for an intraoperative cholangiography was found; hence, the rupture of the intrahepatic bile duct was not confirmed radiologically during surgery. The procedure ran without complications.

**Figure 5:**
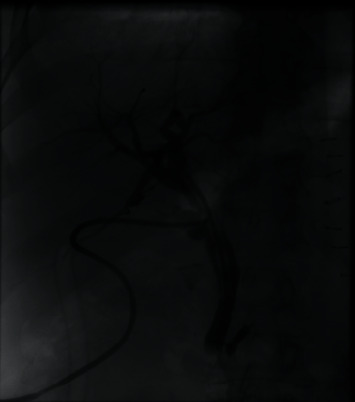
Cholangiography—no aberrant intrahepatic bile ducts are detected.

The postoperative recovery was uneventful. A cholangiography was performed which excluded the presence of any aberrant intrahepatic bile ducts (Figure 5). The patient did not develop jaundice postoperatively. The patient was discharged on POD 15.

## 3. Discussion

The occurrence of duodenal and common bile duct perforations post-ERCP is overall rare (1%) [8], but it is most commonly the cause of biliary peritonitis and pneumoperitoneum post-ERCP [9].

In the context of post-ERCP intestinal perforations, duodenal perforations are regarded as a comparably common post-ERCP complication [10]. The perforations are mostly located in the duodenal wall or perivaterian and are most frequently caused by sphincterotomy or guide wire, less frequently by the endoscope itself or through stent placement [11]. Typical clinical signs of perforation are epigastric and back pain, tenderness with or without signs of peritoneal irritation, but can also be surgical emphysema, tachycardia, and fever [11]. Early diagnosis and prompt treatment is essential in the management of post-ERCP duodenal perforation [12]. Computed tomography has proven to be the most useful imaging method [13]. There are several classifications being used for this condition, but the most frequently used is the Stapfer classification [14], which besides the anatomical localization also includes the mechanism and severity of injury. The treatment can be conservative or surgical, although in recent times, endoscopic treatment has been more and more advocated [15]. The decision depends on the patient's condition, mechanism of injury, and localization of the perforation and degree of its containment [16]. Although, historically, the surgical approach was preferred [17], today, in the majority of cases, conservative treatment is being deployed which consists of nil per mouth, gastric decompression via a nasogastric or nasoduodenal tube, broad spectrum antibiotics, and frequent reevaluation [11]. The surgical management is based on two principles: control of sepsis and perforation repair with or without diversion [18]. Today, the mortality from this condition is around 6% [19] and when compared to earlier literature has almost halved.

On the other hand, the development of intrahepatic bile duct ruptures resulting in biliary peritonitis, and pneumoperitoneum is extremely rare [8]. In literature, the aforementioned condition has been scarcely reported, but its pathophysiology is associated with pathological changes in the liver parenchyma surrounding the ruptured bile duct: liver metastasis and abscesses [6, 20]. There is one case report by Fukui et al. [21] reporting a post-ERCP rupture of an intrahepatic duct, in otherwise, healthy liver parenchyma resulting in biliary peritonitis, but the rupture was associated with an innate anomaly identified as appendix fibrosa hepatis.

In the management of the above reported case, a substantial difference between the radiological and surgical diagnoses was observed. The clinical appearance (acute onset) and CT finding indicated duodenal perforation. The described pneumoperitoneum and especially pneumoretroperitoneum are anatomically associated with duodenal perforation and hence typical signs of it. But, intraoperatively, the duodenum, pancreas, and retroperitoneum were found to be intact, and the ruptured healthy hepatic bile duct was detected. It is curious though how no aberrant subcapsular hepatic bile duct or any other possible anatomic anomaly of the liver was found neither on MRCP, ERCP, or cholangiography. All this implies that our case report is the first in literature to describe a novel case of biliary peritonitis post-ERCP. Our case states that the rupture of a healthy subcapsular hepatic bile duct can lead to biliary peritonitis requiring immediate surgical treatment. The cause of this perforation was probably an excessive air insufflation during ERCP resulting in severe pneumobilia. Hence, the risk for the development of such a perforation cannot be stratified. It can only be recommended to avoid excessive air insufflation during ERCP to prevent this type of complication. Since the rupture of the bile duct in our case occurred probably during or immediately after ERCP, the insertion of an endoscopic nasobiliary drainage duct after ERCP would not have prevented this complication [22]. But our literature review has shown that carbon dioxide insufflation can reduce the incidence of post-ERCP complications such as pneumoperitoneum [23]; hence, its application could be recommended in the prevention of the aforementioned complication in our case.

Our case illustrates the need for a wholesome cooperation and coordination between radiologists and surgeons in the management of post-ERCP complications [9]. First, the radiological findings are essential in deciding between conservative and surgical management [24]. Correctly interpreting CT results enables the differentiation between intestinal perforation and other possible causes of pneumoperitoneum and can be essential for the execution of percutaneous interventions if available [25].

In the treatment of post-ERCP biliary peritonitis, there is no weighing, and immediate surgical treatment is required, no matter the cause being duodenal, common bile duct, or intrahepatic bile duct perforation [26]. In this case, on the other side, the radiological work-up, indicating the perforation site, can help in the planning of the adequate surgical treatment.

Based on our case, we would like to emphasize the diverse etiology of post-ERCP pneumoperitoneum and biliary peritonitis and the importance of good cooperation between radiologists and surgeons treating post-ERCP complications in order to decide for the correct treatment modality, plan adequate surgical procedures, and detect even extremely rare post-ERCP complications which can have fatal consequences [14].

## 4. Conclusions

We report a case of extremely rare etiology of post-ERCP pneumoperitoneum and biliary peritonitis which has never been reported in literature before. Based on the literature review and our case presentation, we conclude that the cooperation between radiologists and surgeon is vital for patients suffering from post-ERCP complications.

## Data Availability

No data were used to support this study.

## Abbreviations

ERCP: Endoscopic retrograde cholangiopancreatography
SCARE: Surgical case report guidelines
AST: Aspartate aminotransferase
ALT: Alanin aminotransferase
IU: International units
CT: Computed tomography
WBC: White blood cell count.
POD: Postoperative day.
